# Cognitive constraints on advance planning of sentence intonation

**DOI:** 10.1371/journal.pone.0259343

**Published:** 2021-11-16

**Authors:** Nele Ots

**Affiliations:** Institute of Linguistics, Wolfgang Goethe University, Frankfurt am Main, Hessen, Germany; CNRS - Université d’Aix-Marseille, FRANCE

## Abstract

Pitch peaks tend to be higher at the beginning of longer than shorter sentences (e.g., ‘A farmer is pulling donkeys’ vs ‘A farmer is pulling a donkey and goat’), whereas pitch valleys at the ends of sentences are rather constant for a given speaker. These data seem to imply that speakers avoid dropping their voice pitch too low by planning the height of sentence-initial pitch peaks prior to speaking. However, the length effect on sentence-initial pitch peaks appears to vary across different types of sentences, speakers and languages. Therefore, the notion that speakers plan sentence intonation in advance due to the limitations in low voice pitch leaves part of the data unexplained. Consequently, this study suggests a complementary cognitive account of length-dependent pitch scaling. In particular, it proposes that the sentence-initial pitch raise in long sentences is related to high demands on mental resources during the early stages of sentence planning. To tap into the cognitive underpinnings of planning sentence intonation, this study adopts the methodology of recording eye movements during a picture description task, as the eye movements are the established approximation of the real-time planning processes. Measures of voice pitch (Fundamental Frequency) and incrementality (eye movements) are used to examine the relationship between (verbal) working memory (WM), incrementality of sentence planning and the height of sentence-initial pitch peaks.

## Introduction

Spoken sentences are characterized by sentence intonation, i.e., modulations of voice pitch. The deviations in voice pitch or, acoustically, changes in Fundamental Frequency (F0) are intentionally controlled (see, e.g., [1–5]) and depend on the linguistic properties of spoken sentences. For example, pitch peaks at the beginnings of spoken sentences are rather high when sentences are long (e.g., ‘The fox that Sidney caught escaped sometime last night from its cage’), but rather low when sentences are short (e.g., ‘The fox has escaped from his cage’) [6–9]. The theory of advance planning of sentence intonation proposes that sentence-initial pitch peaks are planned ahead of articulation because it enables speakers to avoid dropping the voice pitch too low at the end of an utterance [6, 8, 10]. Importantly, the lowest pitch occurs to be rather constant at the ends of spoken sentences for a given speaker [8, 10], indicating, in turn, that speakers are somewhat limited in manipulating the lower register of their voice pitch. While this physiological limitation may represent an important constraint on planning spoken sentences and sentence intonation, it does not explain all of the processes of advance planning of sentence intonation. In particular, several studies find no effect of sentence length on sentence-initial pitch peaks [4, 11, 12]. Thus, as other researchers also note, the study of sentence-initial pitch peaks seems to indicate that the scope of advance planning varies strongly in speakers and in different sentences [4, 8, 11, 13]. This raises the question of what other mechanisms—aside from physiological constraints—might underlie advance planning of sentence intonation.

This study proposes and investigates the scope of (verbal) working memory (WM) in planning spontaneously spoken sentences and sentence intonation. It hypothesizes that sentence-initial pitch peaks depend on the availability of mental resources in the early planning stages. To test the role of WM, the study adopts a dual-task design by combining the picture description task with a recall task where speakers are asked to memorize a list of unrelated nouns before they describe the pictures and recall the words after they have produced the picture description. For investigating the real-time planning of sentences, the experiment records speakers’ eye movements during the picture description task. As such, the study builds on the interference effects known from the study of WM [14, 15] and the notion of incrementality known from the studies of real-time sentence production (tracking of eye movements during a picture description task) [16–21]. As such, the study contributes to the relationship between language production and WM, and develops the cognitive account of advance planning of sentence intonation.

### Impact of mental resources on language production

Producing a spoken sentence that is linked to previous context and listeners’ expectations involves a number of cognitively demanding mental operations related to language planning for production. The production models posit that planning for production proceeds in three stages: conceptualization, linguistic encoding (i.e., formulation) and phonological encoding (i.e., articulation) [22–25]. By rapidly shifting between these stages, speakers convert the intended meaning (message) into abstract-lexical representations (e.g., lemmas), assign syntactic functions appropriate for the message and order the constituents, given the discourse and grammatical constraints. Finally, the stage of phonological encoding activates the word forms and initiates the motor programming in terms of syllable scores for speaking. In other words, psycho-linguistic study of language production has demonstrated that before starting to speak, speakers process linguistic information in different processing stages or mental modules corresponding to the levels of linguistic analysis (phonological, morphological, syntactic and semantic levels) [24, 26–28].

The existing studies of the relationship between the language production and mental resources in terms of WM indicate that limited mental resources influence the three planning stages to varying degrees [14, 15, 29–32]. These studies usually employ dual-task settings, in which speakers are asked to describe pictures and simultaneously remember a list of words, digits or patterns of squares [14, 15, 29–32]. The dual-task design relies on the notion of cognitive load, or the mental effort arising from splitting one’s attention between the two similar tasks [15, 33]. The underlying assumption is that a very similar secondary task will interfere with the main task and hinder the speakers’ linguistic performance (see also discussions in [15, 33]). The effect of interference as approximated by speech onset latencies and error rates is taken to indicate the involvement of WM in language planning for production. The detrimental effect of cognitive load typically means that planning for production becomes more incremental. This means that language chunks devised prior to articulation are rather small, possibly comprising only the first part of a sentence (e.g., ‘A man’ in the sentence ‘A man is pulling a donkey’) [18, 19, 34–38].

While some studies attempt to investigate the impact of cognitive load on formulation and phonological encoding processes, little research has investigated the relationship between WM and conceptual encoding processes. The existing studies indicate that the incrementality of formulation processes does not vary as a function of increasing cognitive load (see, e.g., [14, 15], but also [39]). Phonological encoding, in contrast, is sensitive to the varying degrees of cognitive load such that it tends to become more incremental due to the time pressure or shortage of mental resources [15, 35]. However, Wagner et al. [32] provide mixed findings showing that the incrementality of phonological encoding increases only when WM is taxed with conceptual load (e.g., when speakers need to memorize the size of an object, which decides the complexity of incipient sentences), but it does not increase when WM is taxed with verbal load (e.g., a list of digits). Thus, existing results suggest that the incrementality of phonological, but not linguistic, encoding depends on the availability of mental resources. However, the research on this matter is scarce, providing only few examples of linguistic (or syntactico-semantic) processing [14, 15] and no examples of conceptual processing.

### Advance planning of sentence intonation

The prosodic study of language has established prosodic phrasing and prominence as the core components of sentence intonation. Prosodic phrasing refers to the system of applying acoustic pauses for delimiting semantically and syntactically meaningful units in the continuous stream of speech (for extensive reviews, see [40, 41]). For instance, speakers are known to resolve semantic ambiguities by placing prosodic breaks between the syntactic constituents (e.g., ‘Steve or Sam [pause] and Bob will come’; or ‘Steve or [pause] Sam and Bob will come’) [42, 43]. Prosodic prominence, on the other hand, refers to highlighting certain words or phrases by prosodic means. This means that they are produced longer, louder and with a greater pitch change (see e.g., [44, 45]). One of the most important function of prosodic prominence is highlighting sentence focus [46–49], which evokes links with previous context (see, e.g., [50–52]) and facilitates the rapid processing of reference (see, e.g., [53–57]). As such, prosodic phrasing and prosodic prominence constitute an integral part of spoken sentences. Consequently, a conscious choice of either prosodic pattern or intonation contour makes an important contribution to the intelligibility of speech.

Phonetic research on planning sentence intonation has focussed on the time-related changes in Fundamental Frequency (F0). Namely, the research shows that in declarative sentences, the relative height of F0 maxima (i.e., intonation peaks) usually declines as a function of time [7–9]. Thus, the notion of F0 declination accounts for the fact that the later the intonation peak occurs in an utterance, the lower it is. These studies have also established that the declination rate is slower in longer sentences than it is in shorter sentences (e.g., ‘The fox has escaped from his cage’ vs ‘The fox that Sidney caught escaped sometime last night from his cage’) [7–9]. Even more important, these studies also find that the relative height of F0 maxima at the very beginning of utterances correlates positively to these utterances’ duration [8, 10]. Thus, several studies have taken the length-dependent raising of phrase-initial intonation peaks to index advance planning of sentence intonation because the length-dependent variation of intonation peaks seems to accurately anticipate the ends of utterances (i.e., full clauses) [6, 8, 10, 11].

The fact that sentence intonation must be planned prior to speaking is substantiated by an observation that the lowest F0 at the end of utterances is rather consistent for a given speaker, indicating limitations in the ability to manipulate the lower register of voice pitch [6, 8, 10]. In terms of physiological processes, plentiful evidence shows that linguistically driven F0 maxima and minima correlate only weakly with subglottal air pressure [1–5]. Moreover, the same studies show that laryngeal muscles help in controlling linguistically driven F0 fluctuations [2]. Thus, when controlling the height of sentence-initial intonation peaks and the declination rate, speakers intentionally avoid dropping their voice pitch too low at the end of their utterances.

#### Prosody of cognitive load

Nevertheless, some studies show that the effect of length on sentence-initial pitch peaks may also be absent [4, 11, 12]. For example, in a study of Romance languages, Prieto et al. [11] find the length effect only sporadically and note that individual and dialectal factors influence the occurrence of length-dependent pitch raise in read speech. More importantly, in an investigation of read-aloud German, Fuchs et al. [4] find that the length-dependent pitch raise depends on the prosodic phrasing of spoken sentences. Namely, speakers in their study decided to pause between subject noun phrases and verb phrases (e.g., ‘Lilli-Marlen [pause] ist eine berühmte Frau aus Suhl’, ‘Lilli Marlen [pause] is a famous woman from Suhl.’ vs ‘Lilli-Matthilda Müller [pause] ist eine berühmte Frau aus Suhl’, ‘Lilli Matthilda Müller [pause] is a famous woman from Suhl’), which in turn had a considerable effect on the correlations between the F0 maxima and sentence duration. In particular, the pitch peaks correlated positively with duration of sentence-initial noun phrases, but not with duration of full sentences. Thus, the length effect appeared to interact with prosodic phrasing of these sentences. More generally though, these findings suggest that the physiological constraints on modulating the lower pitch voice are not enough to account for all the mechanisms of advance planning of sentence intonation, or more specifically, the pitch raise at the beginnings of long sentences. Therefore, additional accounts of length-dependent sentence-initial pitch raise are necessary.

Research shows that sentence intonation is sensitive to mental challenges in various metrics indexing loudness, voice quality (e.g., creaky, breathy), speech rate and sentence intonation [58–61]. For example, Lively et al. [60] demonstrate that speakers tasked with tracking a dot on a computer screen while simultaneously speaking, spoke considerably louder, faster and with more monotonous intonation contours (decreased F0 variability) compared to a simple speaking task. Relatedly, Mersbergen and Payne [61] show that sentence intonation, as indexed by average F0, is higher when speakers are asked to read aloud colour names printed in contradicting ink (e.g., a word ‘black’ is printed in blue) than when they read colour names printed in consistent ink (the Stroop task). Thus, in certain contexts, the pitch raise appears to be a tonal response to mental challenges that involve splitting attention between two simultaneous tasks. Somewhat relatedly, the study of foreign language production shows that F0 is higher whilst speaking non-native languages than whilst speaking one’s native language [62–64]. As the second language production is also considered to be mentally more challenging than the first language production [62–64], the pitch raise in non-native language production might similarly approximate the high cognitive demands of language planning. Followingly, these two different lines of research seem to indicate that the pitch height in general but possibly also pitch peaks in particular may depend on the scope of mental (attentional) resources available for the on-going language production.

### The present study

This study aims to investigate the relationship between WM and language production by examining cognitive mechanisms underlying the length-dependent pitch raising in spontaneous speech. It suggests that the relative pitch height at the beginnings of long sentences is related to the extent of mental load involved in the speech production task.

The proposal is that the length effect on sentence-initial intonation peaks indicates mental effort that occurs only when the conceptual or higher-level planning processes are weakly incremental or even non-incremental. The non-incremental conceptual processing entails that a larger network of concepts and relationships between them need to be activated and maintained in the current WM. Relatedly, the number of concepts in long sentences is greater than in short sentences, e.g., consider sentences ‘A farmer is pulling a donkey’ vs ‘A farmer is pulling a donkey and a goat’. Thus, in case of less incremental conceptual processing, the chunk of information concurrently maintained in the WM at the time of manipulating the articulatory information of the first-mentioned word is larger in longer sentences than in shorter sentences. As a result, the sentence-initial pitch peaks might become high as an unintentional (or planned) response to the information load in WM during the production of long sentences. Conversely, pitch is not raised when sentences are short and the number of concepts maintained in WM is rather small.

To demonstrate the impact of WM on the length-dependent pitch raising, this study manipulates the degree of cognitive load with an aim to reduce the number of activated concepts in WM. In particular, the number of concepts is expected to decrease when the cognitive load in the speech production task is high. During the early planning of incipient sentences, the decrease of concepts corresponds to the increase in incrementality of conceptual processing. Thus, the study investigates the interaction between the increasing mental load and the incremental conceptualization processes in advance planning of sentence intonation.

#### Theoretical assumptions of the employed speech production task

We devised an eye-tracking experiment, consisting of two blocks, to verify the proposed cognitive account of advance planning of sentence intonation. The aim of the first block is to establish the tonal differences between short and long sentences when no additional cognitive load is imposed on speech production. For this block, participants are asked to describe monochromatic line drawings depicting an action involving three actors (Fig 2, for similar materials see [65]). Either one or two of the three actors can function as the initiator(s) or the recipient(s) of an action, i.e., agent or patient respectively. In other words, either role can be executed by one or multiple actors. In the case of multiple actors, the two actors may or may not share an identity (e.g., both are donkeys or one is a donkey and the other is a goat).

The concrete task setting ensures that the picture descriptions vary between short and long (e.g., ‘A man is pulling donkeys’ vs ‘A man is pulling a donkey and a goat’). As the length effect is sometimes reported to occur between the sentences consisting of short or long sentence-initial subject noun phrases (e.g., ‘*The man* is pulling the donkey’ vs ‘*The man and the woman* are pulling the donkey’), but to be absent between the sentences containing short or long sentence-final noun phrases (e.g., ‘The man is pulling *donkeys*’ vs ‘The man is pulling *the donkey and the goat*’) [4], the proposed experiment will control the sentence length at both of these syntactic positions. However, the methodological assumption is that the conceptual planning processes do not differ between the two conditions of multiple actors (two agents vs two patients) because the complex visual scenes are similar in that they require an early higher-level conceptual decision for encoding the relationships between the actors depicted [32, 65].

For the investigation of the scope of WM, the second block of the eye-tracking experiment will follow the standard dual-task design, in which speakers are first asked to memorize a list of words, then describe a picture of simple transitive actions, and finally to recall the memorized list of words [14, 15, 29, 31, 35, 66]. Before seeing a picture, speakers are shown three nouns semantically unrelated to the action in the picture and asked to remember these words (e.g., traditsioon, ‘tradition’, otsing, ‘search’, investor, ‘investor’). The conceptual load is tested by the so-called verb probe task, which means that after describing the picture, the participants are prompted with a verb, for which they are requested to orally recall the three nouns and to indicate whether they all fit with the verb on the screen (see procedures in [29] for the modification of the standard WM task to tap the phonological and semantic retention). For example, the nouns ‘tradition’, ‘search’ and ‘investor’ co-occur and are acceptable with a transitive verb ‘jätkama’ in Estonian (according to the frequency counts in the National Estonian Corpus and the previous norming study). Crucially, the verb probe task is expected to tap the conceptual retention and deteriorate the conceptual planning processes in describing the pictures such that speakers spare time on making the higher-level conceptual decisions and proceed with describing the pictures in a more incremental manner. This means that instead of previewing and conceptualizing all actors in the pictures, speakers will preview and conceptualize only the actors they mention first in their picture descriptions.

Contrary to earlier investigations [14, 15], the experiment is designed to elicit spontaneous speech for the two main reasons. First, most of the research on F0 declination relies on read speech [4, 6, 8, 11, 67, 68]. This means that in these studies, speakers usually read aloud written sentences. The fact that the length effect on F0 has been observed in reading only, causes Levelt [24] to cast doubt on advance planning of sentence intonation and the causal relationship between phrase length and phrasal pitch. Some recent studies have addressed this issue by specifically investigating spontaneous speech [10, 13]. Importantly, these studies examine prosodic phrases as defined by the presence of pauses (i.e., speech chunks delimited by acoustic pauses). Notably though, pause-internal speech chunks are in accordance with the notion of incremental planning, as the prosodic edge boundary can be inserted with a fairly small amount of advance planning [24]. Asu et al., [13] also do not find the constant low F0 at the chunk ends, which quite underscores the incremental planning of these prosodic phrases. As the analyses of prosodic phrases extracted from the speech corpora do not record any information about the linguistic context, specific inferences about the incrementality of planning processes cannot be made. Therefore, the existing results might provide a rather one-sided picture of this phenomenon.

Second, earlier investigations often train speakers to use certain lexical items (e.g., names of the pictured actors are learned before experimenting) [14, 15]. Naturally, however, the conceptual processing can most readily occur in speakers who have not yet encountered the concepts and words of interest in the context of a particular experiment. Thus, to examine the most natural conceptual planning processes, the experiment elicits spontaneous utterances in response to pictures. The spontaneous speech production entails large segmental and lexical variation between these sentences, which makes speech onset latencies as standard approximations of cognitive effort less reliable. Moreover, the earlier findings suggest that the stage of conceptual planning involves only about 30% of the time from the picture presentation until the start of speech [17–19, 69]. Therefore, the time between the picture onset and start of an utterance subsumes different stages of planning for production. In turn, the measure of speech onset latency may prove to be somewhat insensitive for diagnosing the interruptions and interferences of cognitive load in the conceptual planning processes.

#### Theoretical assumptions of the new measure of cognitive difficulty

The experiment records speech time-locked to the eye movements in a picture description task to overcome the limitations of measuring speech onset latency. Past research has established this method as reliable for assessing real-time language planning for production [17, 19, 20, 69]. Importantly, many studies since Griffin and Bock [17] have established that some of the stages of sentence planning are clearly observable in the location to which the eye is directed while describing a picture [16–21, 69, 70]. Namely, the picture naming studies have observed that before naming an object, subjects look at an object for a long duration to recall its name, i.e., for lexical and possibly for syntactic processing (e.g., [25]). Similar long viewing times also occur whilst describing pictures in full sentences. Nevertheless, these long viewing times in picture-description tasks appear much later than in picture-naming tasks (e.g., approximately at the 1000 ms from the picture onset). Consequently, while the visual attention is split between the regions of depicted actors to be mentioned in incipient utterances in the first 0 to 400/600 ms of being presented with a picture, it concentrates on the actors in the order of mentioning in later time windows of sentence production [17]. Griffin and Bock [17] argue that this brief time window provides a conceptual frame that guides the subsequent rapid processing of the actors in the order of mentioning [17]. In other words, the eye movements within this early time window of planning index the conceptual planning processes.

Importantly, research shows that the proportions of eye fixations varying as a function of time in this early time window of picture processing are highly sensitive to the linguistic features of incipient utterances. For instance, the proportions of these early eye fixations appear to depend on the word order [20, 69], presence of morphological case-marking [21], syntactic priming [18, 71] and lexical diversity [18]. For example, in a study with Dutch speakers, Konopka and Meyer [18] observed that sentence planning proceeded more or less incrementally depending on how accessible the name of the first-mentioned actor was and on how easy it was to encode the relationships between the depicted actors, i.e., the events. In particular, they found that when the name was easily accessible or when the relational encoding was more difficult, speakers “fell back” on a strictly incremental, that is, word-by-word or concept-by-concept planning strategy [18]. This was indicated by the greater proportions of eye fixations at the first-mentioned actors at around 400 ms of picture presentation. Thus, in the earliest timeframe of picture processing, the increase of the fixation proportions at the first-mentioned actors are taken to indicate more incremental conceptual processing whereas the decrease of the fixation proportions at the first-mentioned actors is taken to indicate less incremental conceptual processing (e.g., hierarchical incrementality) [17, 18, 20, 21, 69, 71]. Consequently, the early and very brief apprehension-phase at the outset of linguistic encoding of visual content is linguistically driven. Furthermore, the eye movements recorded in this early time frame of picture processing approximate the degree of incrementality of conceptual planning processes during real-time sentence production. As such, eye movements constitute an established behavioural measure and a proxy for cognitive processes involved in planning for language production.

The eye movements in the existing studies are typically investigated as continuously changing functions of time in a fixed time window (from 0 to 400/600/1000 ms of picture presentation) [16–21, 69, 70]. For the purposes of this study, we derive a new relative measure from the eye movements based on what is currently known about eye movement in the linguistic task settings [16–21, 25, 69, 70]. The new metric is based on the notion of gaze that is defined as multiple consecutive fixations directed towards the region of interest [72]. As the clear start of phonological processing is indexed by a long and intense gaze towards the first-mentioned actor/object right before the onset of speech, the new measure takes this long, “naming” gaze as a landmark for the time window between the picture onset and the start of an utterance. In other words, the duration of these naming gazes will be divided by the speech onset latencies based on the following reasoning.

Namely, the time before the naming gaze indexes the time window where the conceptual frame of an incipient sentence is generated. The time after this longest gaze indicates encoding of further aspects of the incipient sentence (possibly the verb or second-mentioned actor). The highly incremental processing entails that after the picture onset, speakers start to encode the first-mentioned actors as soon as possible [18, 36, 69]. Since they might also start their speech early, they may spend most of the time period between the picture onset and the start of an utterance with processing the first-mentioned actors. In other words, the duration of the naming gaze at the first-mentioned actor relative to the speech onset latency is comparably high. Conversely, the weakly incremental planning assumes that speakers also take time for processing second-mentioned actors, such that the naming gaze at the first-mentioned actor starts later [16, 19] and the speech onset may be somewhat delayed. This means that the duration of the longest gaze towards the first-mentioned actor relative to the speech onset latency is rather low. Consequently, the degree of incrementality and the effect of cognitive load on conceptual processing can be inferred from the duration of the naming gaze measured proportionally to the speech onset latency. In particular, the increasing proportional duration of the naming gaze at the first-mentioned actor or object approximates the increase of incrementality in the conceptual planning processes.

#### F0 declination in Estonian

This study investigates the impact of WM on conceptual processing and advance planning of sentence intonation based on Estonian. Estonian presents a good test bed for investigating declining intonation contours for three main reasons. First, many researchers have noted that Estonian sentence melody is mostly falling (overview in [73]). In the terminology of the autosegmental-metrical theory of intonation, a low edge tone occurs most frequently at the ends of intonation phrases [74]. Second, pitch accents occurring very regularly on most of the content words are F0 falls (F0 maxima followed by F0 minima; H*+L) [75]. Third, a recent corpus investigation has demonstrated length-dependent F0 raising at the beginnings of prosodic phrases randomly extracted from conversational speech [13]. The fact that the falling pitch accents are most frequent and that they mostly combine with low boundary tones indicates a rather small intonational inventory when compared to fully-fledged intonation languages like English or German. This tonal uniformity is well-suited for measuring F0 maxima in comparable tonal contexts, to replicate the effect of length on sentence-initial intonation peaks, and to examine the effect of cognitive load on the advance planning of sentence intonation.

### Hypotheses

In line with earlier studies, active sentences uttered in response to complex visual scenes are assumed to be produced less rather than more incrementally [15, 32, 35, 65, 76]. Oppermann et al. [76] show for German that sentences with a frequent subject-verb-object ordering (e.g., ‘Die Maus frisst den Käse’, ‘The mouse is eating the cheese’) can be successfully planned in full even at the phonological planning stage. For English, Ferreira and Swets [35] similarly demonstrate that speakers plan their active sentences non-incrementally when no time constraint is imposed. Therefore, this study of Estonian assumes that the conceptual planning under low cognitive load is only weakly incremental or even non-incremental, but it is expected to become more incremental when additional cognitive load is imposed, i.e., under high cognitive load. In other words, if conceptual planning processes share resources with WM, then the detrimental effect of high cognitive load should show increase in the incrementality of conceptual planning. The effect of cognitive load on the incrementality should be observable in the increased proportional duration of the naming gaze at the actor first mentioned before the onset of speech.

**Fig 1:**
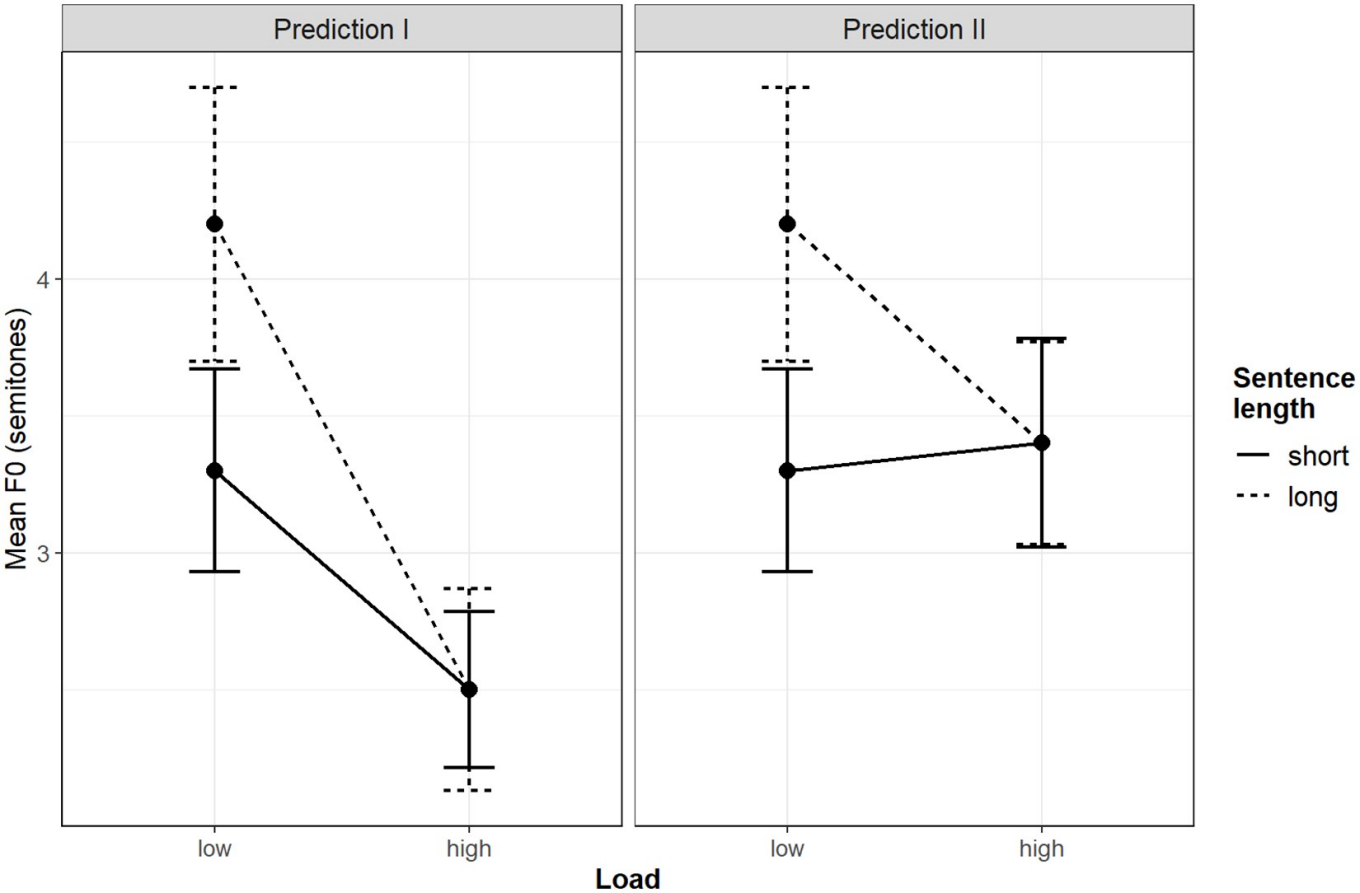
Illustration of hypotheses regarding sentence-initial intonation peaks based on the invented data. Error bars indicate the 95% confidence intervals.

As argued in the previous paragraph, sentence planning is expected to proceed weakly incrementally or even non-incrementally under low cognitive load. Accordingly, tonal variation is expected to be determined by the number of concepts maintained in the WM or by sentence length such that sentence-initial intonation peaks are higher in longer than shorter sentences. The high cognitive load and the incrementality of planning are predicted to be tightly interrelated with the advance planning of sentence intonation. First, the incrementality is expected to relate to low pitch peaks because the highly incremental conceptualization processes only foresee mentioning the first concept in subsequent utterances. Second, the cognitive load is predicted to increase the sentence-initial pitch peaks given that the mental load arising from maintaining a complex conceptual network in WM (as it may occur whilst planning long but not short sentences) is comparable to the mental load that emerges from memorizing a word list. Consequently, two different outcomes of the load manipulation on sentence-initial intonation peaks are possible (see Fig 1).

First, regardless of sentence length, the pitch peaks could decrease to a level lower than the pitch peaks in short sentences under the low cognitive load. In this scenario, the high cognitive load affects short as well as long sentences, and the effect of high cognitive load is greater on long sentences than short sentences. Second, the effects of incrementality and the high cognitive load could accumulate such that, regardless of sentence length, the pitch peaks under the high cognitive load are about the same height as the pitch peaks in the short sentences under the low cognitive load. This scenario predicts the high cognitive load to affect the pitch peaks in long sentences but not short sentences.

## Materials and methods

A picture description experiment was designed to examine the effect of cognitive load on the incrementality of conceptual planning processes, and the effects of cognitive load and the resulting incremental planning processes on sentence-initial intonation peaks. The design and analysis methods are registered prior to collecting data and the Ethics Commission at the University of Tartu approved the experiment (3271T-27).

### Materials

**Fig 2:**
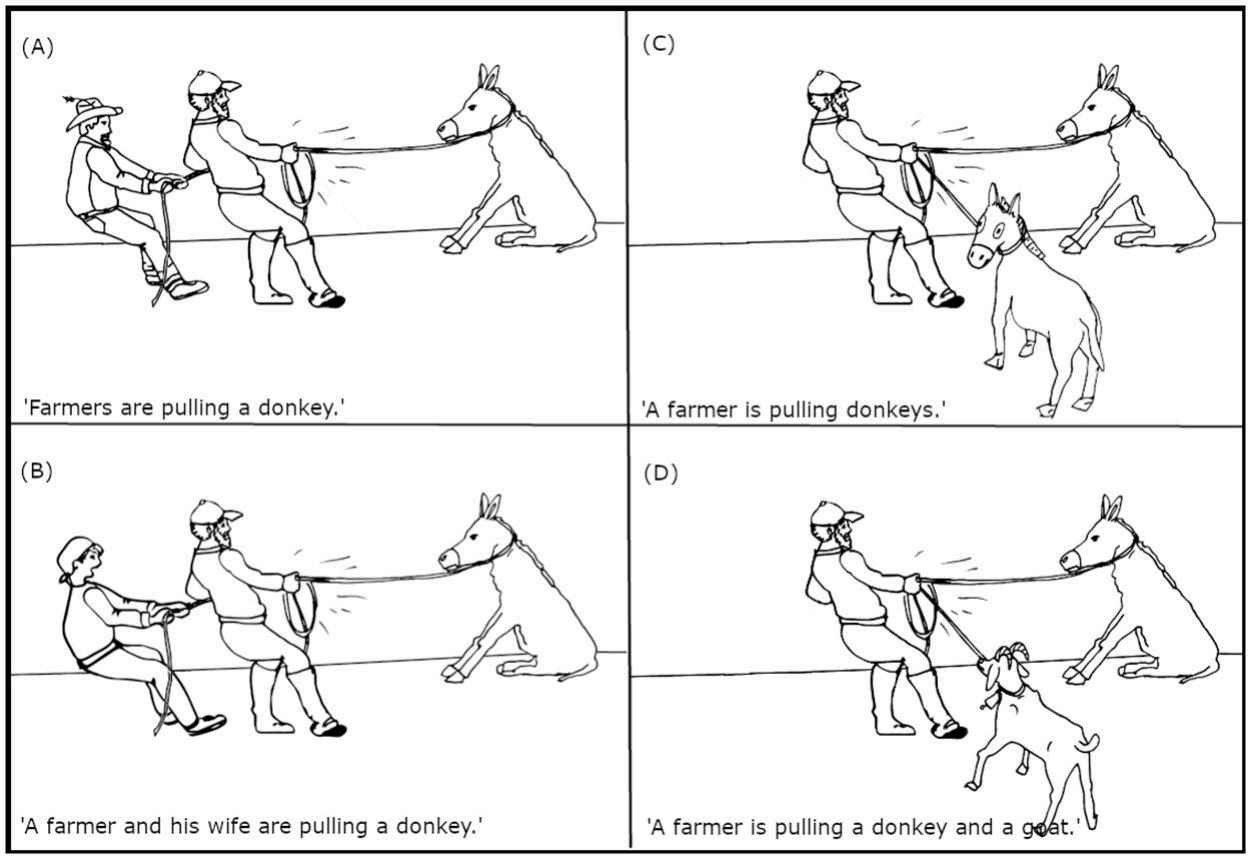
An example picture in four experimental conditions eliciting short and long sentences.

The materials consist of pictures showing transitive events and lists of three unrelated nouns and verbs for the verb probe task. The pictures depict three actors, two of whom share a thematic role. The two core roles of transitive events typically include i) an initiator or agent, and ii) a undergoer of the action or patient. The two thematic roles are distributed amongst the three pictured actors such that either two agents act upon a single patient (Fig 2a and 2b) or a single agent acts upon two patients (Fig 2c and 2d). This constitutes a two-level factor called Double Actor with two levels: agent and patient. A further visual factor that the design manipulates is called Identity. The two actors in the same thematic role either do or do not share their identity (e.g., they are both donkeys or they are a donkey and goat). Thus, the factor Identity includes two levels: same and different. The two visual factors with two levels are fully crossed, resulting in four pictorially controlled conditions. In particular, the four conditions show (i) same multiple agents acting on a single patient (e.g., farmers pulling a donkey), (ii) different multiple agents acting on a single patient (e.g., a male and female farmer pulling a donkey), (iii) a single agent acting on similar multiple patients (e.g., a farmer pulling donkeys), or (iv) a single agent acting on different multiple patients (e.g., a farmer pulling a donkey and goat).

The aim of this fully crossed two-factorial design is to elicit transitive sentences that differ in length, such that the similar double actor condition elicits short sentences and the different double actor condition triggers speakers to produce long sentences. The concrete task setting will ensure that the picture description will indeed be either short or long. In particular, participants are asked to describe the pictures by mentioning all three actors. Moreover, they are trained to use either plural NPs for the actors sharing the identity (e.g., ‘donkeys’) and coordinated NPs for the actors differing in the identity (e.g., ‘a donkey and a goat’).

The complex two-by-two design is necessary for the manipulation of the length effect at the two syntactic positions. This means that either the sentential subjects of prospective sentences are short or long (e.g., Mees sikutab eesleid, ‘The man is pulling the donkey’ vs Mees ja naine sikutavad eesleid, ‘The man and the woman are pulling the donkey’), or the sentential objects vary between short and long (e.g., Mees sikutab eesleid, ‘The man is pulling donkeys’ vs Mees sikutab eeslit ja kitse, ‘The man is pulling the donkey and the goat’). This design allows researchers to control for the possible interactions between the prosodic phrasing and F0 declination [4], and observe the effect of cognitive load in a worst-case scenario where the pitch peaks do not increase together with the length of object NPs.

Altogether, 64 different pictures of simple events (target items) are created for the study (4*64 = 256 pictures in total). Another set of 84 pictures will serve as distractor items. The distractor items will depict a variety of transitive and intransitive events comprising one to four identifiable actors. Half of the pictorial items are combined with a verb probe task separated into a second experimental block.

For the verb probe task, two sets of verbs are selected based on their collocation frequencies in the Estonian National Corpus [77]. The sets of transitive and intransitive verbs are restricted to participate in a moderate numbers of collocations with nouns referring either to animate (marked for subject function) or inanimate entities (morphologically marked for object function). The moderacy restriction (the co-occurrence frequency being between the Leipzig lexical frequency classes of 8 and 12 [78]) is applied for two reasons. First, when the verb participates in a small number of collocations that also occur frequently in the corpus, the verb might aid the recall of the memorized words. Second, and conversely, when the verb participates in a large number of collocations, it may be difficult to delimit the set of nouns that are conventional with these verbs. While the transitive verbs are selected such that they are free to occur with both animate and inanimate referents, the intransitive verbs are determined to encode actions typical for only animate referents.

The memory lists of three nouns are constructed such that they contain a noun that refers to an animate referent and two nouns that refer to inanimate referents. Notably, the variation of animacy in to-be-memorized word lists is regarded as the conceptual aspect of intended messages. The recall task builds on the semantics of verbs. In particular, the lists of nouns (e.g., traditsioon ‘tradition’, otsing ‘search’, investor ‘investor’) are selected such that all nouns are likely to occur with transitive verbs (e.g., jätkama ‘continue’) but only nouns referring to animate referents are conventional with intransitive verbs (e.g., muretsema ‘worry’). Half of the target trials in the second block will be prompted together with transitive verbs and the other half of the trials with intransitive verbs. The correct answers of the verb probe task (whether all of the nouns do conventionally/usually co-occur with the verb in the same sentences or not) have been confirmed in a pre-test with a different sample of native Estonian speakers. To balance the factor of verb type, 50% of distractor trials will be presented with nouns that are incompatible with the transitive verb prompt. Conversely, the other half of the distractor trials will be prompted together with intransitive verbs that are compatible with all of the nouns on a list. Due to the limitations of time and financial resources, the lists and prompts that are combined with distractor items are selected based on the corpus statistics and will not all be pre-tested.

Finally, another set of 12 pictures (practice trials) is comprised of 6 pictures similar to the pictures in target trials and of another 6 pictures similar to the pictures in distractor trials. Similarly, 12 additional pairs of nouns and a verb are constructed for the practice trials.

### Design

The two visual factors Double Actor and Identity, as well as the factor Cognitive Load, all comprising two levels, will be fully crossed, resulting in eight conditions in total. The two-level fixed effect Cognitive Load reflects whether the picture was described while maintaining a list of nouns in WM or not (levels low and high). The trials with no load constitute the first block of the experiment (32 target pictures) and the trials with the load (32 target pictures) constitute the second block.

Four lists of target trials are created by distributing the 256 target pictures between the four conditions (Double Actor crossed with Identity) using the Latin square approach. Target pictures in lists are distributed between the same set of 84 distractor pictures such that there are one or two distractor items for every target item, resulting in 148 experimental trials per list in total. Another set of four lists is created where the leftward placement of agents is reversed, resulting in eight lists. For the verb probe task, all eight lists are reversed for a further set of eight lists, amounting to 16 lists altogether. In all 16 lists, the second half of the trials include the verb probe task.

Participants are presented with all eight conditions across 64 target pictures (repeated measures, within-subject, between-item). The within-subject design is necessary because F0 tends to be highly variable and affected by many other, also non-linguistic, factors. The tonal differences between the conditions can most reliably occur within a speaker, ideally also within the items of the same speaker. Due to this practical limitation, most of the classical intonation research typically records all conditions for a speaker (see e.g., [4, 6–11, 46–49]). The within-item design is not optimal for the purposes of this study, as we aim to record genuine planning processes. Thus, the repeated measures design with within-subject and between-item factor represents the best fit between the purposes of the study and the limitations of the F0 measurements. In other words, the design resembles the ANOVA repeated measures design with a within-subject factor eliciting 64 measurements (8 images X 8 conditions) per participant.

### Participants

Study subjects will be recruited from the university’s students and staff through informational mailing lists at the University of Tartu in Estonia. They must be native speakers of Estonian, and between 18 and 50 years old. The upper age limit is applied because the WM capacities are known to decay in ageing speakers (see in [65]) and due to the COVID-19 pandemic, in which the older participants belong to the higher risk group. Speakers eligible for the study should have normal or corrected to normal vision (soft contact lenses included, glasses excluded), no diagnosed language impairments and normal hearing ability. Subjects will be reimbursed 5 euros for their participation in the study.

Since the experiment is designed such that samples of subjects are going to respond to samples of target trials, PANGEA web application (Power Analysis for General ANOVA designs; [79] provided an *a priori* power analysis. The PANGEA analysis (variation parameters set to PANGEA defaults) indicates that, given the small to medium effect size (d = 0.45), the recommended sample size of 60 subjects is suitable for achieving the statistical power (1-*β*) of 0.8 in the evaluation of the three main effects and the three-way interaction between the fixed effects. Due to the spontaneous nature of the task, not all of the subjects will perform expectedly on all target trials. Therefore, the number of subjects is increased by approximately 33% of observations. Therefore, the resulting sample size is 84 subjects. The estimated sample size is at the limit of the feasibility of planned phonetic analyses and the project’s timeline.

### Procedure

Subjects will be recruited through university mailing lists. The study call will include a registration form that requests subjects to share information about their age, linguistic background, vision and language impairments, if known. Invitations are sent to subjects who conform to the study criteria (see above). Otherwise, the subjects will be thanked for their interest. Together with an invitation letter, subjects will also receive a letter reminding them about their responsibility not to spread any infections, especially COVID-19, and to respect the hygiene rules in the testing rooms.

Upon arriving at the recording room at the University of Tartu, subjects are asked to fill out and sign a consent form informing them about the risks of the study and their right to stop participating in the experiment at any time without any consequences. They will be introduced to the eye tracking method that is carried out with synchronous speech recording.

After these introductory parts, the subjects are asked to seat themselves in front of the desktop-mounted EyeLink 1000 Plus eye tracker and lean their forehead on the head support. The chin is left unsupported because the study requires subjects to speak.

Subjects will be semi-randomly assigned to the 16 lists of experimental trials such that each list will be seen five or six times. The order of the items within a block (low vs high load) is randomized. The items are subdivided into the blocks such that there is the least possibility for semantic overlap between the target and distractor items.

Once the calibration procedures are completed successfully, subjects receive a thorough explanation of their task by seeing a sample of five pictures together with a possible written description in the bottom part of the screen. They will be given an opportunity to ask as many clarification questions as needed.

**Fig 3:**
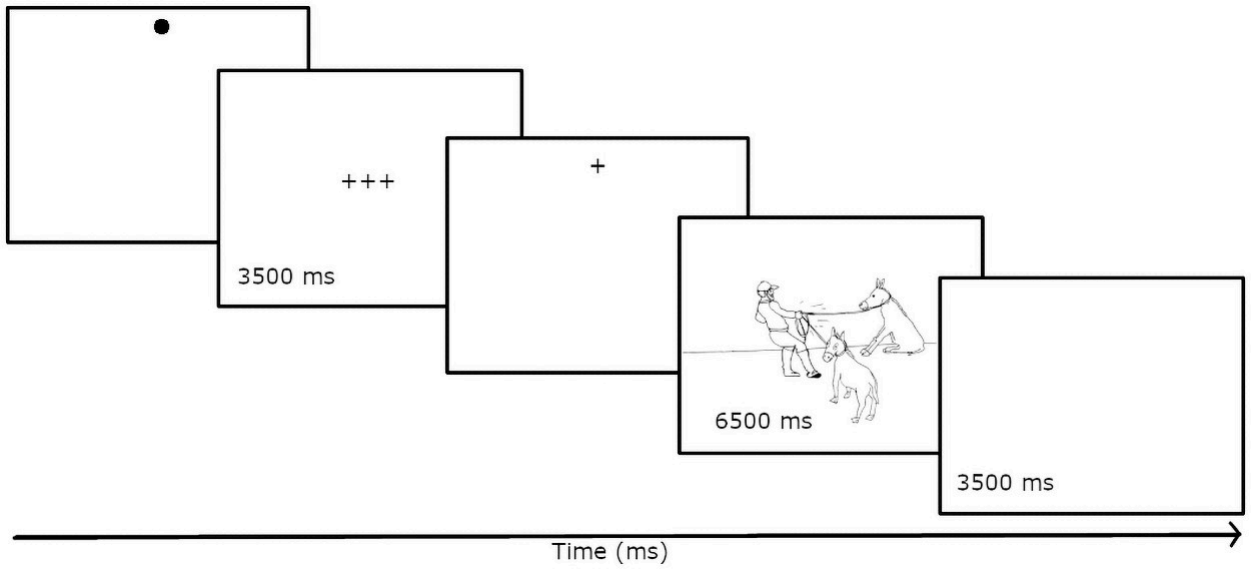
Presentation sequence of stimuli in trials without the concurrent memorization task.

The subjects’ task is to describe simple actions by mentioning all three depicted actors using either plural or coordinated NPs. Every picture is presented for 6500 ms (Fig 3). The speech recording starts at the exact moment that the picture is shown. When the task is clear, subjects will have 6 practice trials to rehearse, after which they can again ask questions. If necessary, subjects are calibrated once more before proceeding to the experiment.

Pictures are presented contingently to gaze. This means that a picture is only shown once the eyes move to the dot in the upper part of the screen. The gaze-contingent presentation ensures that subjects will always look at the dot in the upper part of the screen before seeing the picture. This procedure guarantees that all speakers begin processing of visual information at approximately the same time in all trials. Apart from the drift check, the experiment can proceed without any assistance or control from the experimenter and be interrupted at any point either for a break or quitting.

A break is enforced half-way through the trials to stimulate concentration on the task and to introduce the second block with the trials that are accompanied with the memorization task. First, subjects are presented with 6 practice trials with a task to memorize three words in their exact order and then after 2500 ms, to orally recall the memorized words and then indicate whether all of the memorized words are conventional with the verb on the screen or not. In particular, their task is to say if all of the words fit with the verb on the screen or not by either saying ‘Kõik sobivad’ (They all fit) or ‘Ei sobi’ (Not all words do fit). In the target trials, a correct response to a transitive verb prompt is that all words fit with the verb on the screen. For example, a ‘tradition’ or ‘search’ from the example in the Materials section (traditsioon, ‘tradition’, otsing, ‘search’, investor, ‘investor’) can be ‘continued’ and an ‘investor’ can ‘continue (with)’ something. The correct response to the intransitive verbs is that not all of the words fit. Consider the verb ‘worry’ paired with the nouns ‘tradition’, ‘search’ and ‘investor’ as an example. A ‘tradition’ or ‘search’ cannot ‘worry’ but an ‘investor’ can. Indeed, it is possible to worry about a tradition or search. However, within this particular task setting, this did not occur to our sample of the norming study because the accuracy scores for this word list was 1.0 when presented with the transitive verb ‘continue’ and 0.83 when presented with the intransitive verb ‘worry’. The target items are combined with pairs of nouns and a verb with the highest accuracy scores (see S1 Table).

**Fig 4:**
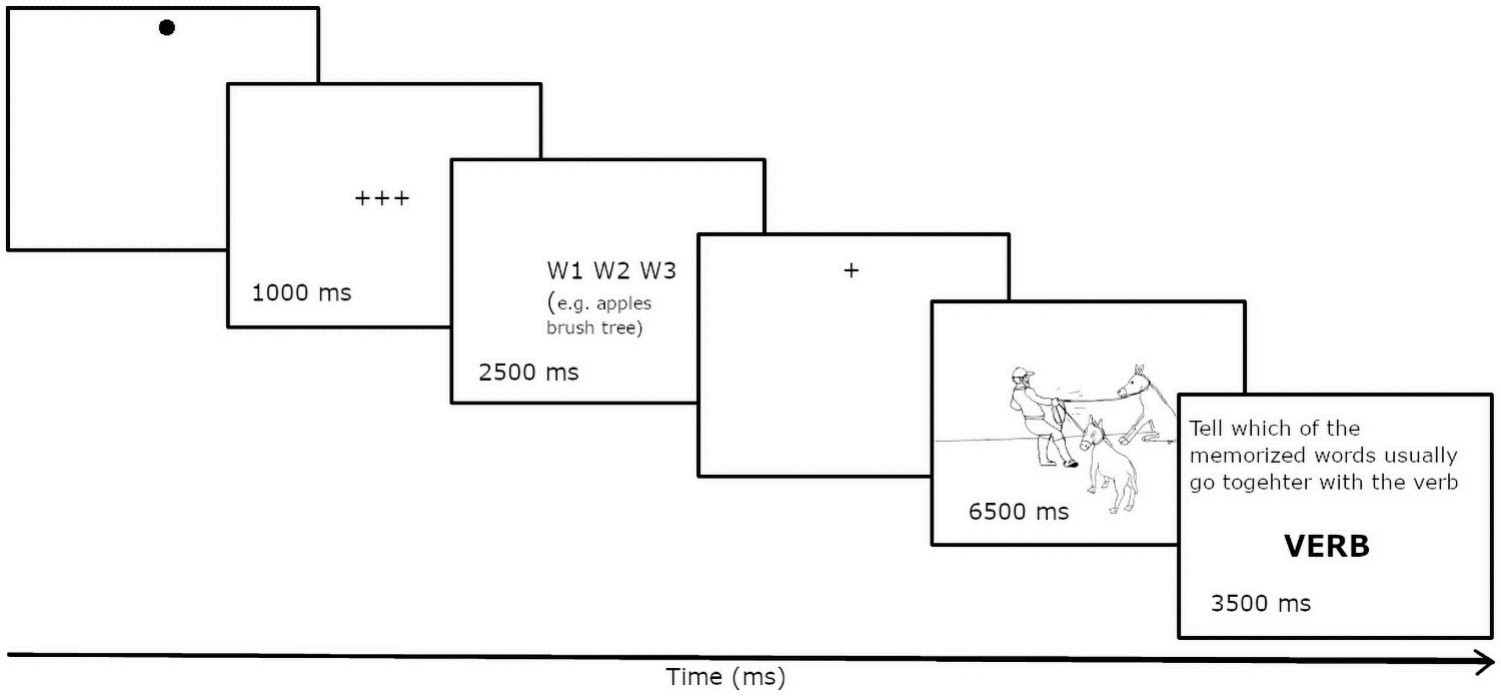
Presentation sequence of stimuli in trials with the concurrent memorization task.

The 6 memory trials are followed with 6 additional practice trials, in which memorization and recall are interposed by a task of picture description. Thereafter, the target and distractor pictures are always preceded by the list of three nouns in the middle of the screen for 2500 ms (Fig 4). Subjects are asked to remember the words in their exact order for later recall. After orally describing a picture, they are prompted with either a transitive or intransitive verb and are asked to recall the memorized words orally and to indicate whether all of the memorized words are conventional with the verb on the screen.

After completing the experiment, subjects are debriefed about the goals of the study, related research and a short list of literature. Together with the debriefing letter, they will receive contact details for future concerns and questions. Upon signing the receipt, they are compensated with 5 euros.

Any personal details collected from subjects during recruiting and experimentation will be stored encrypted for a limited time frame in a password-protected database. All data recorded during the experimentation will be anonymised and analysed separately from the personal details.

### Data analytic plan and proposed analyses

#### Pre-processing and data harvesting

First, starts and ends of utterances will be detected automatically based on the energy levels in the recorded signals. All of the starts and ends are then manually checked in the phonetic analysis software Praat [80], Together with this checking procedure, all descriptions will be manually transcribed and scored for disfluencies (restarts, corrections, hesitations, breaks) and for employed syntactic structures (e.g., transitive, intransitive, truncated passive). Based on these transcriptions, onsets and offsets of word labels are then determined with the help of the forced alignment tool provided by the Bavarian Archive for Speech Signals (BAS) [81]. The automatic annotation of speech segments is again manually checked with special attention to silent and filled pauses, as they determine the boundaries of prosodic phrases. Finally, all this information is created and stored for each sentence in the TextGrid format provided in Praat. Most importantly, the start of utterances will serve as the measure of speech onset latencies.

F0 of each sentence (in hertz) is extracted with the help of the autocorrelation method available in Praat in two passes. During the first pass, F0 tracks are extracted with default settings for the lowest and highest F0, the “floor” and “ceiling” (75 Hz and 600 Hz, respectively). Then, the first and third quartiles of F0 (Q1 and Q3) are calculated for each speaker and recorded in a table. In the second pass, F0 contours are extracted with speaker-specific settings (0.75*Q1 for floor and 1.5*Q3 for ceiling). The F0 samples making up a contour are saved in the two-column format where the first column contains timestamps and the second column F0 measures at given times.

The timing and position of eye fixations is recorded relative to the regions of depicted actors, the so-called areas of interest (AOI). The AOIs are determined on the basis of the thematic roles. This means that AOIs are defined to cover the areas of agents and patients such that the actors sharing a thematic role are enclosed into a single AOI.

For linguistic scoring, all sentences will be tagged for a number of linguistic parameters including the syntactic structure, number of actors mentioned, names of the actors, correspondence to the intended actors and mentions of any additional noun phrases or sentence modifiers. In addition, the length of sentences and syllable scores of sentence constituents and actor names are extracted from the phonetic annotations contained in the TextGrids.

A number of exclusion criteria are defined based on the eye movements, linguistic parameters, speech onset latencies and the accuracy of the verb probe task. These exclusions are necessary for the comparability between the sentences because the conceptual planning processes during language production are tightly related to speech timing and the syntactic structures employed. Therefore, target trials will be excluded when the first fixation to the AOI occurs later than 400 ms after picture onset, or when the time delay between the two consecutive fixations is longer than 600 ms (indication of track loss). Picture descriptions of a subject will be included only when (s)he is able to remember at least one word correctly in 90% of the load trials. The final analyses will only include fluent transitive sentences. After these exclusions, additional trials will be excluded based on speech onset latencies later than three standard deviations above the grand mean. Finally, all the data from the subject will be removed when the subject’s inclusion rate after the indicated exclusions is lower than 25%. No treatment of missing data is necessary because the planned regression modelling method (LMER) [82–84] is robust to unbalanced data.

#### Dependent variables

The dependent variables of interest are (i) speech onset latencies, (ii) duration of naming gazes, (iii) duration of naming gazes proportional to their respective speech onset latencies, (iv) the height of sentence-initial pitch peaks as indexed by F0 maxima (v) and the height of sentence-final pitch valleys as indexed by F0 minima.

**Fig 5:**
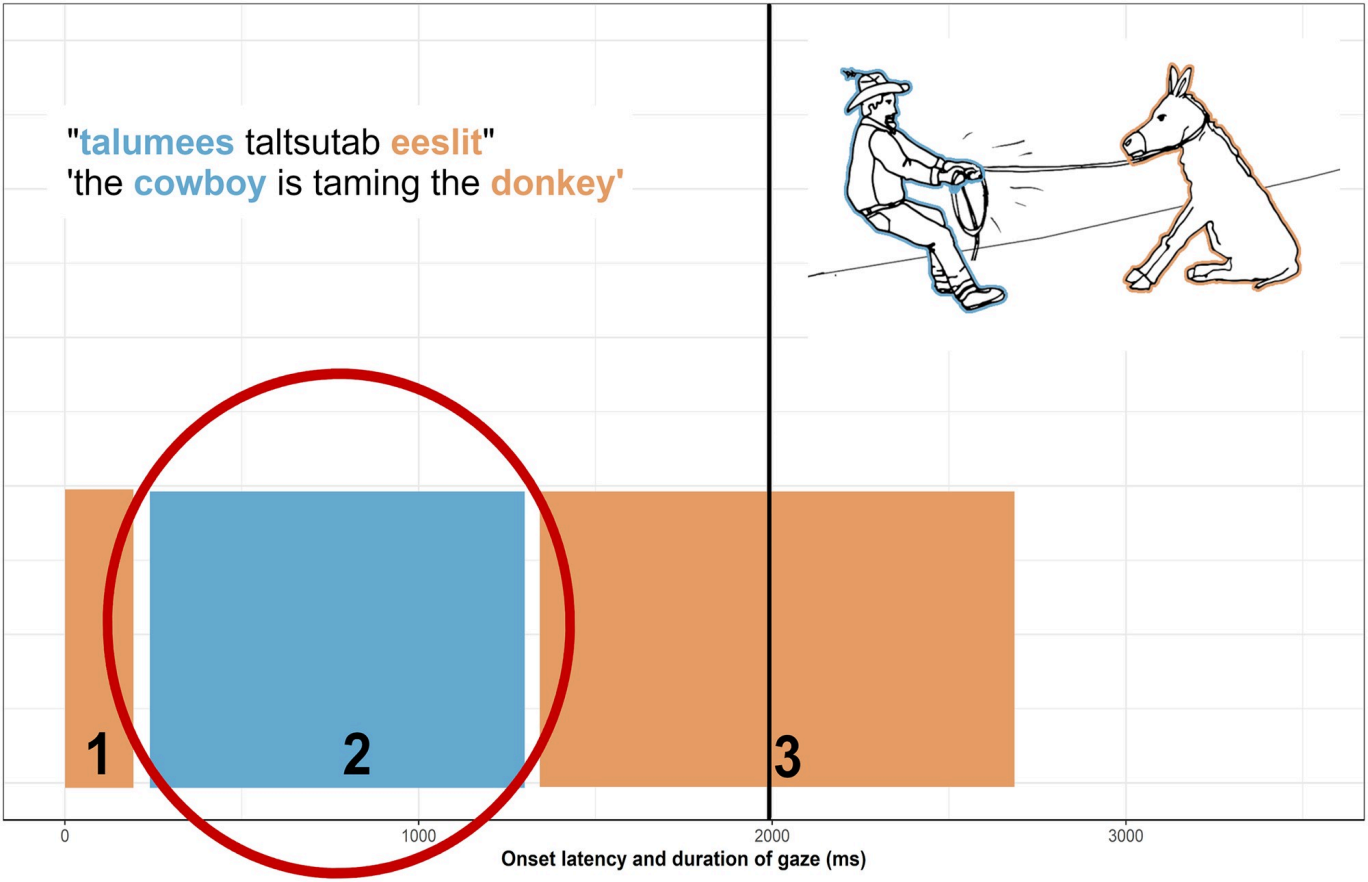
Hypothetical onset latencies (left edge of bars) and duration (widths of bars) of gazes directed to a particular area of interest (e.g., blue refers to the agent initiating and orange to the patient undergoing the action of pulling) during the description of an item ‘man pulling a donkey’ in Estonian. The numbers on the squares count the gazes. The black vertical line indicates the speech onset latency. The red ellipse highlights the gaze that is called ‘naming gaze’ and defined as a gaze of longest duration prior to the start of an utterance. For assessing the degree of incrementality, the duration of the naming gaze is divided by the speech onset latency (for similar analysis procedures and data see, e.g., [19]).

First, the starts of utterances (in milliseconds) index the speech onset latencies. Second, a gaze is defined as multiple consecutive fixations directed at the AOI [72]. Gaze duration is calculated by subtracting the onset time of the first fixation directed at the AOI from the offset time of the last consecutive fixation directed to the AOI. Typically, before the speech onset, between one and five gazes occur (see Fig 5). The number of gazes usually increases together with the complexity of the visual scene. The data analysis algorithm will compare between the durations of the gazes occurring before the speech onsets and determine the gaze with the maximum duration, i.e., the longest gaze. The naming gaze is reported as missing (NA) when, in the very unlikely case of only one gaze or of two gazes of exactly the same duration, the algorithm cannot detect a gaze with the maximum duration. The longest gaze before the speech onset is called the naming gaze and submitted to further analyses together with its AOI (either agent or patient).

Finally, F0 maxima and F0 minima are automatically extracted within the boundaries of agent and patient names and the boundaries of sentence-final words in cases where patient names do not occur at the end of a sentence (e.g., some sentence modifier (e.g., varsti, ‘soon’) follows the patient name). F0 maxima and minima measured in hertz (Hz) will be converted into semitones relative to speaker means based on Eq (1)

$$\begin{array}{c} F0_{st}=12*log_{2}\left(\frac{F0_{Hz}}{F0_{mean}}\right), \end{array}$$ (1)

where *F*0_*mean*_ is the subject’s mean F0 aggregated over all F0 contours extracted from all of the subject’s utterances. This method of converting F0 into semitones has the benefit of partly cancelling out the large-scale, gender-based differences in voice pitch (males typically have lower voice pitch than females). The effect of sentence length on sentence-initial pitch peaks is expected to be smaller than the gender effect. When gender variation in voice pitch is included in the data, the critical effects of sentence length and WM may remain unrevealed.

#### Statistical analysis

The goal of the statistical analyses is to determine whether the scaling of sentence-initial pitch peaks in long sentences is affected by an independent effect of incrementality or by the simultaneous effects of incrementality and high mental load. As the manipulations of the factors Double Actor (agent vs patient) and Identity (same vs different) elicit short and long sentences (see in Materials), the combinations of these are reduced into a factor Sentence Length (short vs long) for the main analysis. Thus, the main analysis examines the effect of the interaction between Cognitive Load (low vs high) and Sentence Length (short vs long) on (i) the proportional duration of naming gazes at the first-mentioned actors, (ii) duration of naming gazes, (iii) the sentence-final pitch valleys, (iv) and the sentence-initial pitch peaks.

The four dependent variables (duration of naming gazes, proportional gaze durations, sentence-final F0 minima, sentence-initial F0 maxima) are modelled with separate linear mixed-effects regression analyses (LMER, as implemented in the *lme4* package in [82, 85]). All models include the interaction between Cognitive Load (low vs high) and Sentence Length (short vs long) as the fixed effect, the block and trial number as the covariate effects, and random intercepts and slopes for items and subjects. The analysis will be carried out using sum-to-zero coding of the fixed effects. To avoid the convergence issues, the models will be optimized following the procedures suggested in Bates et al. [82]. First, the continuous variables block and trial are centred and/or scaled for the statistical analyses. Second, the convergence tolerances are adjusted by applying an optimizing algorithm diverging from the standard test but which results in the converging model. Third, and only in case the previous measures do not yield the convergence of the model fit, the random effects structure will be simplified by removing the correlations term from the random effects structure. Finally, the significances of the model estimates are obtained with the help of the *lmerTest* package [86] in R [85]. The significances of the main effects and the interaction are determined at the threshold of p<0.05.

The first analysis tests the extent to which the proposed verb probe task tapped the conceptual retention. The proportional duration of naming gazes at the first-mentioned actors is assumed to increase under the high cognitive load. Thus, only the main effect of cognitive load is expected in this analysis. The second analysis investigates the absolute duration of naming gazes, as the interference effect may arise at the phonological planning stage (or semantico-syntactic encoding) instead. In particular, if the phonological encoding of the first-mentioned actors is disrupted due to the memorization of three words the duration of naming gazes right before the speech onsets should increase under the high cognitive load.

The third analysis checks the preliminaries of advance planning of sentence intonation. Namely, the aim is to test how constant the sentence-final low F0 is in the elicited sentences. For this, the statistical evaluation should find no effect of Sentence Length. Nevertheless, the sentence-final low F0 may increase under the high cognitive load, as the existing studies have reported generally higher and flatter F0 contours for speech under mentally challenging conditions [58–64].

Finally, the fourth analysis tests the effect of cognitive load on the scaling of sentence-initial pitch peaks. The significant interaction between Cognitive Load and Sentence Length should demonstrate that the degree of length-dependent pitch raise in sentence-initial pitch peaks depends on the cognitive load. If the cognitive load causes sentence planning processes to proceed incrementally and the incrementality of planning processes mainly bears on the scaling of the pitch peaks, then the sentence-initial pitch peaks are lower under the high load than under the low load, and the difference between the high and low load is greater in long sentences than in short sentences. If the incrementality and the high load both play a role in pitch scaling, then the tonal difference between the high and low cognitive load is expected to occur only in long sentences.

The exploratory analyses inspect the three-way interaction between the factors Double Actor (agent vs patient), Identity (same vs different) and Load (low vs high) on (i) the proportional duration of naming gazes at the first-mentioned actors (ii) and the sentence-initial pitch peaks. The first exploratory analysis of naming gazes aims to determine whether the pictorial scenes combining the similar and differing event participants either in agent or patient role would induce planning increments differing and gradually increasing in size. Known from the studies relying on the grouping effect (though with moving displays), the chunk conceptualized and planned before the speech onset increases together with the more complex phrase structures [37, 38]. The second analysis will assess whether the length-dependent pitch raising depends on the syntactic position of the length variation (as in [4]). If a large number of sentences will be uttered with a break between the subject noun phrase and the verb phrase, the desired significant interaction with Identity (i.e., Sentence Length) and Cognitive Load might only occur when Double Actor corresponds to the agent. The models of the exploratory analyses and the significance testing will follow the same procedures as described for the main analyses.

#### Publication of data

All data except the speech recordings will be made available on the Open Science Foundation’s data sharing platform. As the voice characteristics constitute identifying data, the speech recordings will remain unpublished. Nevertheless, the database will provide the records of eye movements (raw and processed), TextGrids with acoustic analyses, tables with F0 samples, spreadsheets with the linguistic parameters of elicited sentences and the analysis scripts.

### Proposed timeline

We aim to begin collecting data in July 2021 and finish in October 2021. Student assistants from Tartu (Estonia) will help to conduct the acoustic analyses. Given the amount of manual work that speech recordings require, the data analyses can be completed by January 2023 at the earliest. Following the completion of data collection, the data analyses and discussions will be finalized and documented by March 2022.

## Supporting information

S1 Table Results of the norming study where each noun was presented together with a verb (e.g., traditsioon + jätkama, ‘tradition + continue’, otsing + jätkama, ‘search + continue’, investor + jätkama, ‘investor + continue’) and participants were asked to say whether all the nouns fit with the verb. Two experiments were necessary because the first experiment did not deliver enough suitable items for the main experiment.
(TEX)

## References

[1] Atkinson JE. Correlation analysis of the physiological factors controlling fundamental voice frequency. The Journal of the Acoustical Society of America. 1978;63(1):211–222. doi: 10.1121/1.381716 632414

[2] Honda K. Physiological factors causing tonal characteristics of speech: from global to local prosody. In: Speech Prosody. Nara, Japan; 2004.

[3] Honda K, Hirai H, Masaki S, Shimada Y. Role of Vertical Larynx Movement and Cervical Lordosis in F0 Control. Language and Speech. 1999;42(4):401–411. doi: 10.1177/00238309990420040301 10845244

[4] Fuchs S, Petrone C, Krivokapić J, PhilipHoole. Acoustic and respiratory evidence for utterance planning in German. Journal of Phonetics. 2013;41:29–47. doi: 10.1016/j.wocn.2012.08.007

[5] Strik H, Boves L. Control of fundamental frequency, intensity and voice quality in speech. Journal of Phonetics. 1992;20(1):15–25. doi: 10.1016/S0095-4470(19)30250-5

[6] Cooper WE, Sorensen JM. Fundamental Frequency in Sentence Production. Springer-Verlag; 1981.

[7] Hart Jt, Collier R, Cohen A. A Perceptual Study of Intonation: An Experimental-Phonetic Approach to Speech Melody. Cambridge Studies in Speech Science and Communication. Cambridge University Press; 1990.

[8] Liberman M, Pierrehumbert J. Intonational Invariance under Changes in Pitch Range and Length. In: Aronoff M, Oehrle RT, editors. Language Sound Structure. Studies in Phonology. Presented to Morris Halle by his Teacher and Students. Cambridge, MA: The Massachusetts Institute of Technology; 1984. p. 155–233.

[9] Maeda S. A characterization of American English intonation; 1976.

[10] Yuan J, Liberman M. F0 declination in English and Mandarin Broadcast News Speech. Speech Communication. 2014;65:67–74. doi: 10.1016/j.specom.2014.06.001

[11] Prieto P, D’Imperio M, Elordieta G, Frota S, Vigário M. Evidence for’soft’ preplanning in tonal production: Initial scaling in Romance. In: Speech Prosody, May 2006, Dresden, Germany; 2006. p. 803–806.

[12] Tøndering J. Preplanning of intonation in spontaneous versus read aloud speech: evidence from Danish. In: Lee WS, Zee E, editors. Proceedings of the 17th International Congress of Phonetic Sciences; 2011. p. 2010–2013.

[13] Asu EL, Lippus P, Salveste N, Sahkai H. F0 declination in spontaneous Estonian: implications for pitch-related preplanning. In: Proceedings of Speech Prosody, Boston 31 May–3 June 2016; 2016.

[14] Ivanova I, Ferreira VS. The role of working memory for syntactic formulation in language production. Journal of Experimental Psychology: Learning, Memory, and Cognition. 2018; 10.1037/xlm0000672 30589334 PMC6707904

[15] Klaus J, Mädebach A, Oppermann F, Jescheniak JD. Planning sentences while doing other things at the same time: effects of concurrent verbal and visuospatial working memory load. Quarterly Journal of Experimental Psychology. 2017;70(4):811–831. doi: 10.1080/17470218.2016.1167926 26985697

[16] Bock K, Irwin DE, Davidson DJ. Putting first things first. In: Henderson J, Ferreira F, editors. The interface of language, vision, and action: Eye movements and the visual world. New York, NY, US: Psychology Press; 2004. p. 249–278.

[17] Griffin ZM, Bock K. What the eyes say about speaking. Psychological Science. 2000;11:274–279. doi: 10.1111/1467-9280.00255 11273384 PMC5536117

[18] Konopka AE, Meyer AS. Priming sentence planning. Cognitive Psychology. 2014;73:1–40. doi: 10.1016/j.cogpsych.2014.04.001 24838190

[19] Kuchinsky SE, Bock K, Irwin DE. Reversing the hands of time: changing the mapping from seeing to saying. Journal of experimental psychology Learning, memory, and cognition. 2011;37(3):748–756. doi: 10.1037/a0022637 21534707 PMC3087166

[20] Norcliffe E, Konopka AE, Brown P, Levinson SC. Word order affects the time course of sentence formulation in Tzeltal. Language, Cognition and Neuroscience. 2015;30(9):1187–1208. doi: 10.1080/23273798.2015.1006238

[21] Sauppe S, Norcliffe E, Konopka AE, Valin, Jr RDV, Levinson SC. Dependencies First: Eye Tracking Evidence from Sentence Production in Tagalog. In: Knauff M, Pauen M, Sebanz N, Wachsmuth I, editors. Proceedings of the 35th Annual Meeting of the Cognitive Science Society (CogSci 2013). Austin, TX: Cognitive Science Society; 2013. p. 1265–1270.

[22] Garrett MF. The Analysis of Sentence Production. vol. 9 of Psychology of Learning and Motivation. Academic Press; 1975. p. 133–177.

[23] Garrett MF. Levels of processing in sentence production. In: Butterworth B, editor. Language production. London: Academic Press; 1980. p. 177–220.

[24] Levelt WJM. Speaking: From intention to articulation. Cambridge, MA: MIT Press; 1989.

[25] Levelt WJM, Schriefers H, Vorberg D, Meyer AS, Pechmann T, Havinga J. The time course of lexical access in speech production: A study of picture naming. Psychological Review. 1991;98:122–142. doi: 10.1037/0033-295X.98.1.122

[26] Bock K, Levelt W. Language production. Grammatical encoding. In: Gernsbacher MA, editor. Handbook of Psycholinguistics. San Diego: Academic Press; 1994. p. 945–984.

[27] Dell GS, Chang F, Griffin ZM. Connectionist Models of Language Production: Lexical Access and Grammatical Encoding. Cognitive Science. 1999;23(4):517–542. doi: 10.1207/s15516709cog2304_6

[28] Levelt WJM, Roelofs A, Meyer AS. A theory of lexical access in speech production. The Behavioral and brain sciences. 1999;22:1–38; discussion 38–75. doi: 10.1017/S0140525X99001776 11301520

[29] Martin RC, Yan H, Schnur TT. Working memory and planning during sentence production. Acta Psychologica. 2014;152:120–132. doi: 10.1016/j.actpsy.2014.08.006 25216074

[30] Martin R, Slevc LRC. Language production and working memory. In: Goldrick VF, Miozzo M, editors. Oxford library of psychology. The Oxford handbook of language production. Oxford University Press; 2014. p. 437–450.

[31] Slevc LR. Saying what’s on your mind: working memory effects on sentence production. Journal of experimental psychology Learning, memory, and cognition. 2011;37 6:1503–14. doi: 10.1037/a0024350 21767058 PMC3199029

[32] Wagner V, Jescheniak JD, Schriefers H. On the flexibility of grammatical advance planning during sentence production: Effects of cognitive load on multiple lexical access. Journal of experimental psychology Learning, memory, and cognition. 2010;36:423–40. doi: 10.1037/a0018619 20192540

[33] Barthel M, Sauppe S. Speech Planning at Turn Transitions in Dialog Is Associated With Increased Processing Load. Cogn Sci. 2019;43(7):e12768. doi: 10.1111/cogs.12768 31310021

[34] Bock JK, Ferreira VS. Syntactically speaking. In: The Oxford handbook of language. Oxford, England: Oxford University Press; 2014. p. 21–46.

[35] Ferreira F, Swets B. How incremental is language production? Evidence from the production of utterances requiring the computation of arithmetic sums. Journal of Memory and Language. 2002;46:57–84. doi: 10.1006/jmla.2001.2797

[36] Gleitman LR, January D, Nappa R, Trueswell JC. On the give and take between event apprehension and utterance formulation. Journal of Memory and Language. 2007;57(544–596). doi: 10.1016/j.jml.2007.01.007 18978929 PMC2151743

[37] Wheeldon L, Ohlson N, Ashby A, Gator S. Lexical availability and grammatical encoding scope during spoken sentence production. Quarterly journal of experimental psychology (2006). 2013;66:1653–73. doi: 10.1080/17470218.2012.754913 23286440

[38] Wheeldon L, Smith M. Phrase structure priming: A short-lived effect. Language and Cognitive Processes. 2003;18(4):431–442. doi: 10.1080/01690960244000063

[39] Power MJ. Sentence production and working memory. The Quarterly Journal of Experimental Psychology. 1985;37:367–385. doi: 10.1080/14640748508400940

[40] Cutler A, Dahan D, van Donselaar W. Prosody In the Comprehension of Spoken Language: A Literature Review. Language and Speech,. 1997;40(2):141–201. doi: 10.1177/002383099704000203 9509577

[41] Wagner M, Watson DG. Experimental and theoretical advances in prosody: A review. Language and cognitive processes. 2010;25(22096264):905–945. doi: 10.1080/01690961003589492 22096264 PMC3216045

[42] Lehiste I. Phonetic disambigation of syntactic ambiguity. Glossa. 1973;7:107–122.

[43] Petrone C, Truckenbrodt H, Wellmann C, Holzgrefe-Lang J, Wartenburger I, Höhle B. Prosodic boundary cues in German: Evidence from the production and perception of bracketed lists. Journal of Phonetics. 2017;61:71–92. doi: 10.1016/j.wocn.2017.01.002

[44] Beckman ME, Pierrehumbert JB. Intonational structure in Japanese and English. Phonology Yearbook. 1986;3:255–309. doi: 10.1017/S095267570000066X

[45] Campbell N, Beckman ME. Stress, Prominence, and Spectral Tilt. In: Intonation: Theory, Models and Applications–Proceedings of an ESCA Workshop; 1998. p. 67–70.

[46] Breen M, Fedorenko E, Wagner M, Gibson E. Acoustic correlates of information structure. Language and cognitive processes. 2010;25(7):1044–1098. doi: 10.1080/01690965.2010.504378

[47] Cooper W, Eady SJ, Mueller PR. Acoustical aspects of contrastive stress in question-answer contexts. Journal of Acoustical Society of America. 1985;77:2142–2155. doi: 10.1121/1.392372 4019901

[48] Féry C, Kügler F. Pitch accent scaling on given, new and focused constituents in German. Journal of Phonetics. 2008;36:680–703. doi: 10.1016/j.wocn.2008.05.001

[49] Swerts M, Krahmer E, Avesani C. Prosodic marking of information status in Dutch and Italian: a comparative analysis. Journal of Phonetics. 2002;30:629–654. doi: 10.1006/jpho.2002.0178

[50] Birch S, Clifton JC. Focus, accent, and argument structure: Effects on language comprehension. Language and Speech. 1995;38(4):365–391. doi: 10.1177/002383099503800403 8816086

[51] Bock JK, Mazzella JR. Intonational marking of given and new information: some consequences for comprehension. Memory and Cognition. 1983;11:64–76. doi: 10.3758/BF03197663 6855561

[52] Toepel U, Pannekamp A, Alter K. Catching the news: Processing strategies in listening to dialogs as measured by ERPs. Behavioral and Brain Functions. 2007;53(3). doi: 10.1186/1744-9081-3-53 17922900 PMC2098771

[53] Cutler A, Foss DJ. On the role of sentence stress in sentence processing. Language and Speech. 1977;20:1–10. doi: 10.1177/002383097702000101 592948

[54] Dahan D, Tanenhaus MK, Chambers CG. Accent and reference resolution in spoken-language comprehension. Journal of Memory and Language. 2002;47:292–314. doi: 10.1016/S0749-596X(02)00001-3

[55] Mehta G, Cutler A. Detection of target phonemes in spontaneous and read speech. Language and Speech. 1988;31:135–156. doi: 10.1177/002383098803100203 3256770

[56] Watson DG, Arnold JE, Tanenhaus MK. Tic Tac TOE: Effects of predictability and importance on acoustic prominence in language production. Cognition. 2008;106(3):1548–1557. doi: 10.1016/j.cognition.2007.06.009 17697675 PMC2274964

[57] Weber A, Braun B, Crocker MW. Finding referents in time: eye-tracking evidence for the role of contrastive accents. Language and speech. 2006;49(3):367–392. doi: 10.1177/00238309060490030301 17225671

[58] Christodoulides G. Effects of cognitive load on speech production and perception [phdthesis]. Université catholique de Louvain; 2016.

[59] Huttunen K, Keränen H, Väyrynen E, Pääkkönen R, Leino T. Effect of cognitive load on speech prosody in aviation: Evidence from military simulator flights. Applied Ergonomics. 2011;42(2):348–357. doi: 10.1016/j.apergo.2010.08.005 20832770

[60] Lively SE, Pisoni DB, Van Summers W, Bernacki RH. Effects of cognitive workload on speech production: Acoustic analyses and perceptual consequences. The Journal of the Acoustical Society of America. 1993;93(5):2962–2973. doi: 10.1121/1.405815 8315159 PMC3499954

[61] van Mersbergen M, Payne AE. Cognitive, Emotional, and Social Influences on Voice Production Elicited by Three Different Stroop Tasks. Folia Phoniatr Logop. 2020. 10.1159/000508572 32668434

[62] Järvinen K. Voice characteristics in speaking a foreign language. A study of voice in Finnish and English as L1 and L2. Tampere: Tampere University Press; 2017.

[63] Järvinen K, Laukkanen AM. Vocal loading in speaking a foreign language. Folia Phoniatrica et Logopaedica. 2015; p. 1–7. 25925665 10.1159/000381183

[64] Peters J, Frank JM, Rohloff M. Pitch range variation in High German (L1) and Low German (L2). In: Proceedings of the 10th Conference on Speech Prosody, May 25, 2020, Tokyo, Japan (virtual conference). 231; 2020.

[65] Hardy SM, Wheeldon L, Segaert K. Structural priming is determined by global syntax rather than internal phrasal structure: Evidence from young and older adults. Journal of experimental psychology Learning, memory, and cognition. 2020;46:720–740. doi: 10.1037/xlm0000754 31545625

[66] Ishkhanyan B, Boye K, Mogensen J. The Meeting Point: Where Language Production and Working Memory Share Resources. Journal of psycholinguistic research. 2019;48:61–79. doi: 10.1007/s10936-018-9589-0 29882117

[67] Ladd DR. Declination “reset” and the hierarchical organization of utterances. The Journal of the Acoustical Society of America. 1988;84:530–544. doi: 10.1121/1.396830

[68] Thorsen NG. A study of the perception of sentence intonation –Evidence from Danish. The Journal of the Acoustical Society of America. 1980;3(67):1014–1030. doi: 10.1121/1.384069 7358909

[69] Sauppe S. Word Order and Voice Influence the Timing of Verb Planning in German Sentence Production. Frontiers in Psychology. 2017;8:1648. doi: 10.3389/fpsyg.2017.01648 29018379 PMC5623055

[70] Griffin ZM. Gaze durations during speech reflect word selection and phonological encoding. Cognition. 2001;82:B1–B14. doi: 10.1016/S0010-0277(01)00138-X 11672707 PMC5130081

[71] van de Velde M, Meyer AS, Konopka AE. Message formulation and structural assembly: Describing “easy” and “hard” events with preferred and dispreferred syntactic structures. Journal of Memory and Language. 2014;71(1):124–144. doi: 10.1016/j.jml.2013.11.001

[72] Griffin ZM, Davison JC. A technical introduction to using speakers’ eye movements to study language. In: Jarema G, Libben G, Westbury C, editors. Methodological and Analytic Frontiers in Lexical Research (Part II). 1; 2011. p. 53–82. doi: 10.1075/ml.6.1.03gri

[73] Asu EL. The phonetics and phonology of Estonian intonation [phdthesis]. University of Cambridge; 2004.

[74] Asu EL. Towards a phonological model of Estonian intonation. In: Langemets M, Penjam P, editors. Proceedings of the Second Baltic Conference on Human Language Technologies, Tallinn 4–5 May 2005. Tallinn: Tallinn University of Technology and Institute of the Estonian Language; 2005. p. 95–100.

[75] Asu EL, Nolan F. The effect of intonation on pitch cues to the Estonian quantity contrast. In: Ohala J, editor. Proceedings of the 14th International Congress of Phonetic Sciences. San Francisco: University of California; 1999. p. 1873–1876.

[76] Oppermann F, Jescheniak JD, Schriefers H. Phonological advance planning in sentence production. Journal of Memory and Language. 2010;63(4):526–540. doi: 10.1016/j.jml.2010.07.004

[77] Kallas J, Koppel K. Eesti keele ühendkorpus 2019. Center of Estonian Language Resources; 2020. Available from: 10.15155/3-00-0000-0000-0000-08565L.

[78] Brysbaert M, Buchmeier M, Conrad M, Jacobs AM, Bölte J, Böhl A. The word frequency effect: a review of recent developments and implications for the choice of frequency estimates in German. Experimental psychology. 2011;58:412–24. doi: 10.1027/1618-3169/a000123 21768069

[79] Westfall J, Kenny DA, Judd CM. Statistical power and optimal design in experiments in which samples of participants respond to samples of stimuli. Journal of Experimental Psychology: General. 2014;143(5):2020–45. doi: 10.1037/xge0000014 25111580

[80] Boersma P, Weenink D. Praat: Doing phonetics by computer [Computer program]; 2020. Available from: Version 6.1.09, retrieved 26 January 2020 from http://www.praat.org/.

[81] Kisler T, Reichel U, Schiel F. Multilingual processing of speech via web services. Computer Speech & Language. 2017;45:326–347. doi: 10.1016/j.csl.2017.01.005

[82] Bates D, Mächler M, Bolker B, Walker S. Fitting Linear Mixed-Effects Models Using lme4. Journal of Statistical Software. 2015;67(1):1–48. doi: 10.18637/jss.v067.i01

[83] Wood SN. Stable and Efficient Multiple Smoothing Parameter Estimation for Generalized Additive Models. Journal of the American Statistical Association. 2004;99(467):673–686. doi: 10.1198/016214504000000980

[84] Wood SN. Generalized Additive Models: An Introduction with R. 2nd ed. Chapman and Hall/CRC; 2017.

[85] R Core Team. R: A Language and Environment for Statistical Computing; 2019. Available from: https://www.R-project.org/.

[86] Kuznetsova A, Brockhoff PB, Christensen RHB. lmerTest Package: Tests in Linear Mixed Effects Models. Journal of Statistical Software. 2017;82(13):1–26. doi: 10.18637/jss.v082.i13

